# Simple Device to Measure Pressure Using the Stress Impedance Effect of Amorphous Soft Magnetic Thin Film

**DOI:** 10.3390/mi11070649

**Published:** 2020-06-30

**Authors:** Joerg Froemel, Satoru Akita, Shuji Tanaka

**Affiliations:** 1 Advanced Institute for Materials Research, Tohoku University, 2-1-1 Katahira, Aoba-ku, Sendai 980-8577, Japan; 2 Department of Robotics, Tohoku University, 6-6-01, Aza Aoba, Aramaki Aoba-ku, Sendai 980-8579, Japan; akita@mems.mech.tohoku.ac.jp (S.A.); tanaka@mems.mech.tohoku.ac.jp (S.T.)

**Keywords:** magnetic thin film, pressure sensing, stress impedance effect, micro device

## Abstract

A simple micro-machined pressure sensor, based on the stress-impedance (SI) effect, was fabricated herein using typical micro-fabrication technologies. To sense pressure, a 1-µm thin, soft magnetic metallic film of FeSiB was sputtered and used as a diaphragm. Its electrical response (impedance change) was measured under pressure in a frequency band from 5 to 500 MHz. A lumped-element equivalent electric circuit was used to separate the impedance of the soft magnetic metal from other parasitic elements. The impedance change clearly depended on the applied pressure. It was also shown that the impedance change could be explained by a change in relative permeability, according to the theory of the SI effect. The radial stress in the diaphragm and the relative permeability exhibited a linear relationship. At a measurement frequency of 200 MHz, the largest sensor response, with a gauge factor of 385.7, was found. It was in the same order as the conventional sensors. As the proposed device is very simple, it has the potential for application as a cheap pressure sensor.

## 1. Introduction

The permeability, *µ*, of soft ferromagnetic materials is influenced by perturbations, such as magnetic field, temperature and strain. The change in permeability can be understood by the resulting change in impedance, *Z*, which is measured with an alternating current. This is due to the skin effect. An alternating current in a conductor creates an alternating magnetic field. Due to the change in the magnetic field, an electric field that opposes the change in current intensity is created. Thus, the electrons of the current are forced out of the center of the conductor, where the field is strongest [1]. The current density *J* in the conductor follows this distribution:

(1) $$J=J_{S}e^{-\left(1+j\right)\frac{z}{\delta }}$$

The current density at the surface of the conductor is *J*_S_, *z* is the distance from the surface and *δ* is the skin depth. *δ* is defined as the depth at which *J* has reached 1/e of *J*_S_ and can be approximated as follows:

(2) $$\delta =\sqrt{\frac{2\rho }{\omega \mu }}$$

where *ρ* is the resistivity of the conductor, *ω* is the angular frequency of the current, and

(3) $$\mu =\mu_{r}\mu_{0}$$

where *µ*_r_ is the relative permeability of the conductor material, and *µ*_0_ is the permeability in a vacuum. *δ* can be understood as the effective conductive thickness. A low permeability results in a large skin depth, resulting in low impedance; a high permeability yields high impedance accordingly.

The dependence of the complex impedance of a magnetic conductor on the magnetic field is called the magneto-impedance (MI) effect [2,3,4,5]. This effect is strong and is widely used to create highly sensitive magnetic field sensors [6,7,8,9]. It was discovered that the MI effect in particular, and the magneto-elastic properties in general, depended on the strain (stress) of the material [10,11]. The inverse magnetostrictive effect, also known as the Villari effect, is responsible for a change in the magnetic anisotropy that influences the permeability by applied stress [12]. There are several models to describe this stress-impedance (SI) effect [13,14]. This effect has been used to create strain gauges based on wires, ribbons, and thin films [15,16,17,18,19,20]. One way to characterize such sensors is the gauge factor (*GF*):

(4) $$GF=\frac{\frac{∆Z}{Z}}{\varepsilon }$$

where *∆Z* is the impedance change induced by strain *ε*. It is possible to achieve gauge factors of more than 1000 [20]. In a configuration where a high-permeability material surrounds a non-magnetic conductor, the transducer effect is large [21]. In the case of a thin-film device, typically a sandwich of a non-magnetic highly conductive layer between soft ferromagnetic layers, e.g., FeSiB/Cu/FeSiB or CoSiB/Cu/CoSiB, with a total thickness in the range of several µm is used [22,23]. As for applications other than as a strain gauge, the possibility of using it as a force sensor, for structural health monitoring, or a pressure sensor has been mentioned [24,25]. Nevertheless, no actual pressure measuring device, using this effect, has been presented yet. Here, we present a study of a simple micro-fabricated pressure sensor using thin-film technology based on the SI effect. It demonstrates the first practical use of the SI effect in a pressure sensor. In contrast to conventional piezoresistive pressure sensors, its fabrication technology is very simple.

## 2. Experimental Section

Most of the traditional micro-fabricated pressure sensors consist of a diaphragm with integrated semiconductor piezoresistive strain gauges that are arranged in, for example, a Wheatstone bridge configuration. Although a similar setup can be constructed by replacing the semiconductor strain gauges with SI thin-film strain gauges, we wanted to demonstrate the feasibility of a sensor with a very simple fabrication and design and without the need for many process steps.

**Figure 1:**
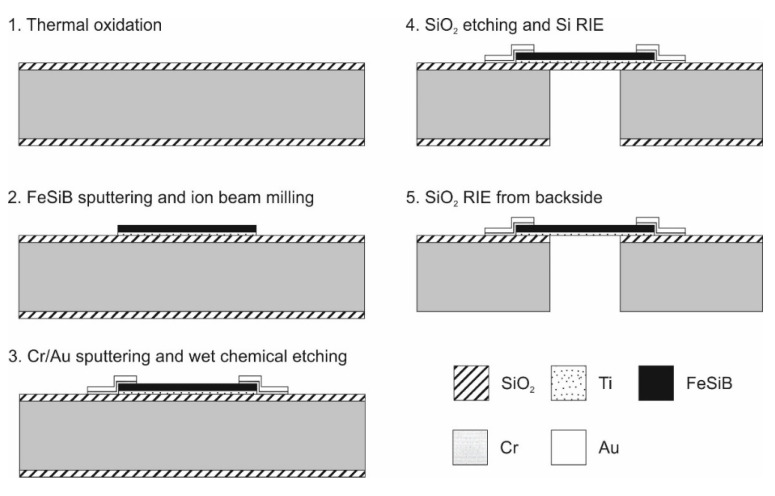
Process flow of the fabrication of the pressure sensor—shown by its cross-section.

The device was designed as a circle diaphragm with a diameter of 1 mm on a silicon substrate. The whole diaphragm was constituted of FeSiB that had no additional structure on it. Electric contacts were made at the sides of the diaphragm to allow the impedance across the whole diaphragm to be measured. A schematic of the fabrication process is summarized in Figure 1.

A 100-mm double-sided polished Si wafer (300 µm thick) was used as a substrate. The process began with the deposition of 1-µm-thick thermal SiO_2_. This layer worked as an electrical insulator and a mask for silicon backside etching. A functional layer of FeSiB was formed with a thickness of 1 µm after a Ti adhesion layer with a 20-nm thickness had been deposited by sputtering. Instead of sputtering from an alloy target, the equipment allowed for the simultaneous sputtering from the four targets used (ULVAC, QAM-4-S). By choosing the power of each target’s magnetron, the composition of the sputtered film could be precisely controlled. After measuring the sputter rate of each target, the sputter power was individually adjusted. The composition was confirmed by inductively coupled plasma mass spectrometry (ICP–MS). To achieve the desired composition of Fe_79_Si_7_B_14_, three targets—Fe, FeB (50:50 at%), and Si—were sputtered at 150, 75, and 150 W, respectively. The total deposition rate of the obtained film was 9.3 nm/min, and the ICP–MS measurement showed a composition of Fe_78.88_Si_6.54_B_14.58_. To reduce the intrinsic stress of the non-deflected diaphragm as much as possible, the sputter process was optimized. The stress of a sputtered thin film depends on many parameters, such as film thickness, substrate temperature, and sputter rate. In this case, the Ar flow rate was chosen as the optimization parameter, and all other conditions left unchanged.

**Figure 2:**
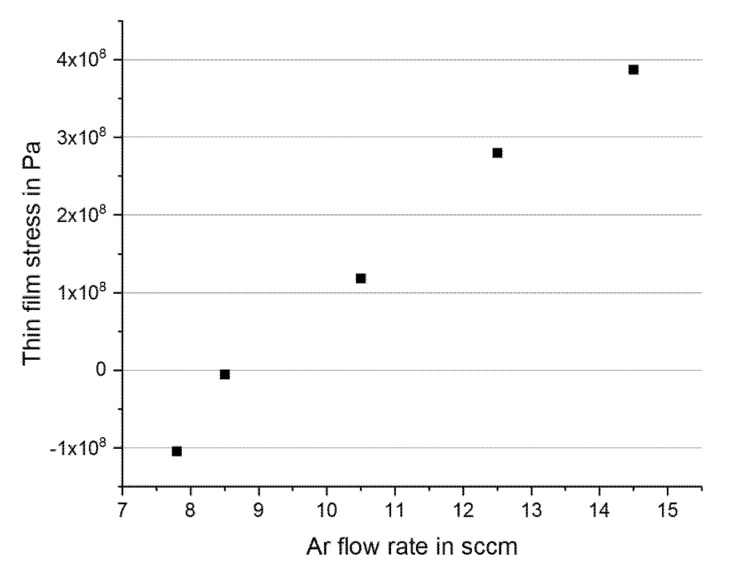
Intrinsic stress of the sputtered FeSiB against the Ar flow rate during sputtering.

The lowest stress was obtained as −5 MPa at an Ar flow rate of 8.5 sccm (Figure 2). Below 7.8 sccm and above 15.5 sccm, no stable plasma could be maintained at the Fe target. The amorphous nature of the film was confirmed using X-ray diffraction (XRD D8 Advance, Bruker, Billerica, MA, USA). The metal film was etched by ion-beam milling. OFPR-800 200cp (Tokyo Ohka Kogyo, Tokyo, Japan) with a thickness of 3 µm was used as a photoresist mask. Next, Au electrodes were deposited. RF magnetron sputtering (CSF-4ES-232, Shibaura Mechatronics, Yokohama, Japan) was employed to deposit a 300-nm-thick blanket Au layer, with a 20-nm-thick Cr adhesion layer. Cr was used because it has good adhesion to both SiO_2_ and FeSiB. Photolithography using OFPR-800 was again conducted on the blanket Au layer. The electrodes were then patterned by wet etching using aqueous iodine solution (KI + I_2_). The photoresist was stripped after the etching process. Next, the SiO_2_ on the backside was etched to form a mask for the Si reactive ion etching (RIE). Through the Bosch process (MUC-21, SPT, Tokyo, Japan), the diaphragm, which formed the core part of the pressure sensor, was opened from the backside. After that, the mask material on the backside and the isolation layer below the diaphragm, both of which were thermal SiO_2_, were etched by RIE. During the etching, the front side was protected by photoresist. We observed no distortion of the membrane after completing the fabrication process. XRD analysis also showed that the amorphous nature of the FeSiB metal did not change as a result of the fabrication steps.

**Figure 3:**
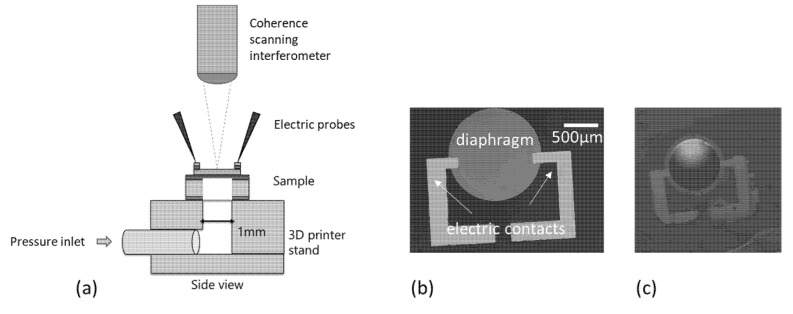
(**a**) Schematic setup to characterize the pressure sensor. (**b**) Microscopic image of a diaphragm and its contacts. (**c**) Picture of a device with deformed diaphragm during measurement.

To characterize the diaphragm, the setup shown in Figure 3 was prepared. It consisted of a 3D-printed stand where the demonstrator was mounted on top and an Ar gas source was connected via a tube. The pressure in the device was controlled by a PC via a microcontroller. The setup allowed for the observation of the device under test conditions from the top using a coherence scanning interferometer (Nexview, Zygo, Middlefield, CT, USA).

**Figure 4:**
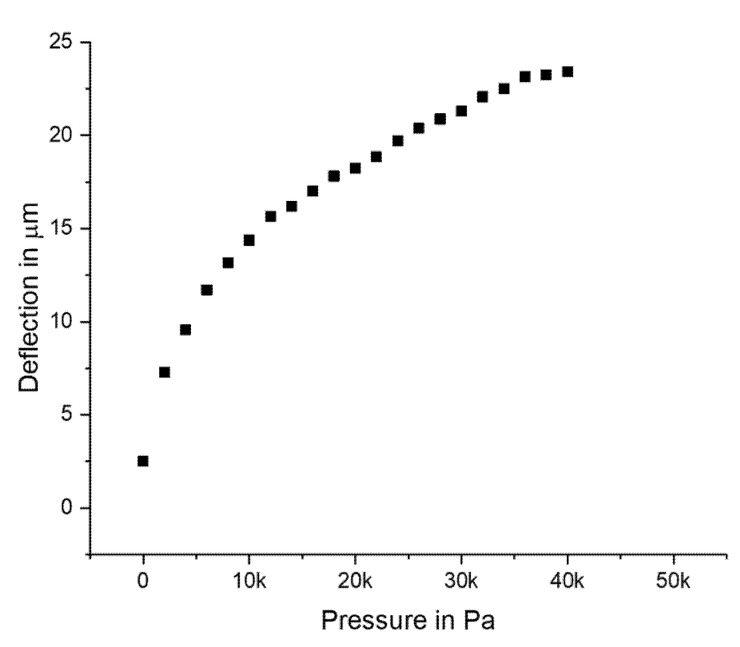
Measured deflection of the sensor diaphragm vs. applied pressure.

The deformation of the device was observed upon application of different pressures from the backside of the diaphragm—From 0 to 40 kPa in steps of 2000 Pa. Figure 4 shows the deflection vs. pressure. It followed the expected nonlinear behavior for a fully clamped circular diaphragm with large deflection according to [26]. The setup allowed for a maximum of 40 kPa to be applied. The diaphragm did not break in a test at maximum pressure.

The impedance of the device was measured by a network analyzer (E5071B, Agilent, Santa Clara, CA, USA) using a high-frequency (HF) probe station in the range from 5 to 500 MHz.

## 3. Results

**Figure 5:**
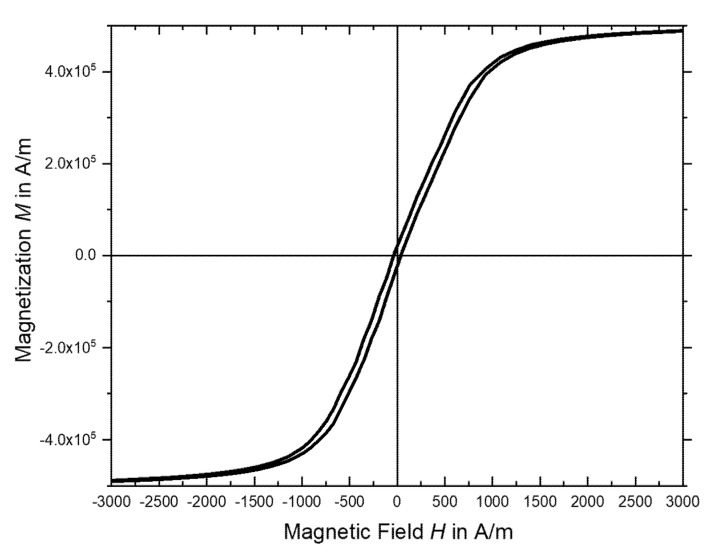
Hysteresis loop of the FeSiB thin film.

The basic electric and magnetic properties of the sputtered FeSiB film were measured. I-V characteristics were linear and the resistivity was determined by the 4-point probe method to be 1.65 µΩm. A superconducting quantum interference device (SQUID) was used to measure the in-plane direction magnetic hysteresis curve of the thin film at room temperature (Figure 5). The parameters derived from the measurements are summarized in Table 1.

The sputtered material clearly showed soft magnetic characteristics. The values were comparable with the films obtained by other groups [2,27].

**Figure 6:**
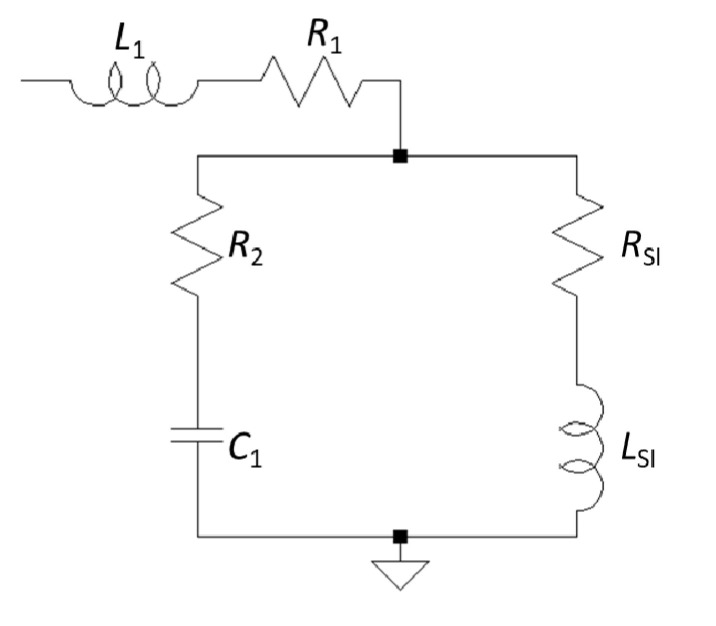
Equivalent circuit of the device under test. *R*_1_, *R*_2_, *L*_1_, and *C*_1_ are parasitic elements; *R_SI_* and *L_SI_* are the transducer elements of the diaphragm.

A simple lumped equivalent circuit was used to model the electrical properties of the device. This model was useful to separate the effect of the parasitic elements of the device under test from the effect caused by stress that was induced in the diaphragm due to deformation by the pressure difference. The equivalent circuit consists of two serial branches, one of which consists of two parallel branches, as shown in Figure 6.

The series branch of *L*_1_ and *R*_1_ represents the parasitic effect of the contact paths to the diaphragm. *C*_1_ is caused by the capacitive feedthrough via the substrate, and *R*_2_ represents the resistance of the substrate and non-moveable FeSiB. The soft magnetic transducer consists of *R_SI_* and *L_SI_*. *R_SI_* is determined by considering the skin effect, and, in the case of a wide, very thin strip, it can be approximated as:

(5) $$R_{SI}=R_{DC}\left(1+\frac{1}{48}{\left(\frac{t}{\delta }\right)}^{4}\right)\;$$

where *R_DC_* is the *DC* resistance and *t* is the thickness of the film [28]. The skin depth *δ* is given by Equation (2). *L_SI_* is caused by the self-inductance of the transducer material. In the case of a thin strip with a length *l* and width *w* >> thickness, it can be calculated as follows:

(6) $$L_{SI}=\frac{\mu_{0}\mu_{r}}{6\pi w^{2}}\left[3w^{2}l\ln\frac{l+\sqrt{l^{2}+w^{2}}}{w}-{\left(l^{2}+w^{2}\right)}^{\frac{3}{2}}+3wl^{2}\ln\frac{w+\sqrt{l^{2}+w^{2}}}{l}+l^{3}+w^{3}\right]$$

The impedance *Z_SI_* is the impedance of the diaphragm metal:

(7) $$Z_{SI}=R_{SI}+\omega L_{SI}$$

Per Equations (2), (5), and (6) [29], it can be seen that *R_SI_* and *L_SI_*, and therefore *Z_SI_*, depend on the relative magnetic permeability *µ*_r_ of the transducer metal.

**Figure 7:**
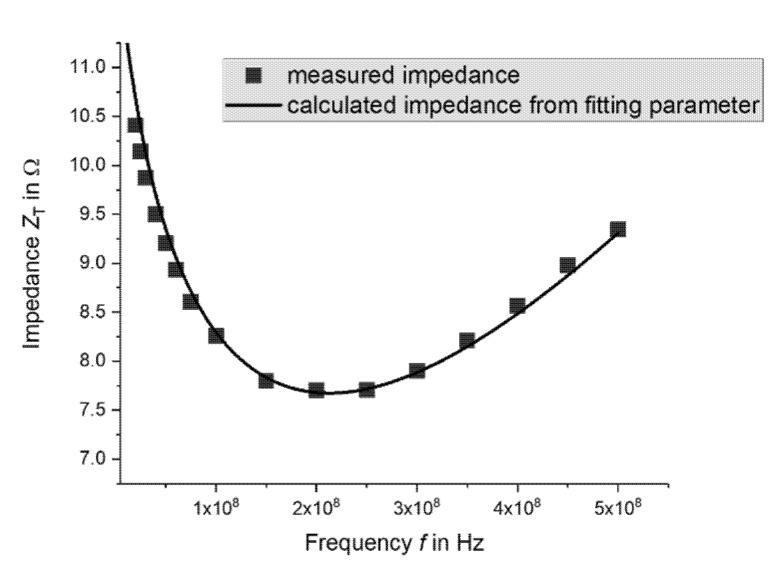
Measured frequency characteristic of the total impedance *Z*_T_ of the device and impedance characteristic calculated from the lumped sum equivalent circuit obtained by fitting.

To obtain the values of *R*_1_, *R*_2_, *L*_1_, and *C*_1_, the total impedance *Z*_T_ of the device was measured between 5 and 500 MHz, without inducing any deformation of the diaphragm using pressure. The total impedance *Z*_T_ of the device varied between 7.7 and 10.4 Ω, as shown in Figure 7.

The values of the equivalent circuit elements were determined by fitting them with the target of minimizing the error Δ between the measured impedance *Z*_T_ and the calculated impedance *Z*_Calc_, which is given as follows:

(8) $$∆={\left(Z_{T}-Z_{\mathrm{Calc}}\right)}^{2}$$

As the starting values of the fitting, the values estimated from the dimensions of the device were used. The Levenberg–Marquart method was employed as an algorithm to minimize Δ. The results with the best fitting are shown in Table 2. The obtained values are reasonable for the parasitics of a device of this size. A comparison between the frequency characteristics of *Z*_T_ and the frequency characteristics obtained by calculation with the equivalent circuit shows a good agreement (Figure 7).

The relative permeability *µ*_r_ of the soft magnetic film under this condition was also obtained as 2306 through fitting.

**Figure 8:**
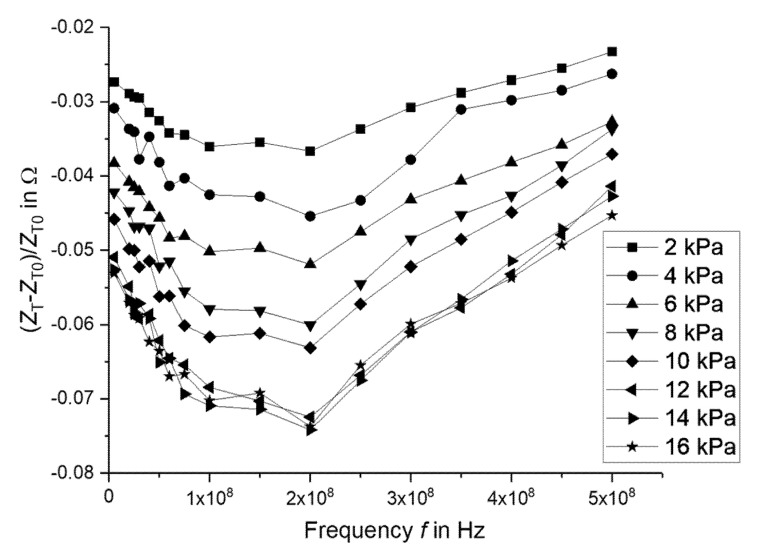
Frequency characteristic of the magnitude of the impedance for different applied pressures to the device under test.

Figure 8 shows the change in the impedance of the device under different applied pressures. Over the entire frequency range, the impedance decreased with increasing pressure. The effect was the strongest at 200 MHz.

**Figure 9:**
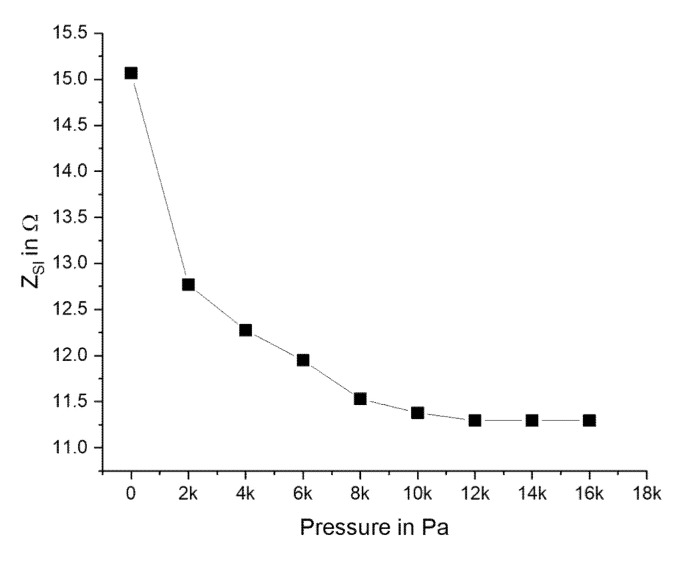
Derived magnitude of the impedance *Z*_SI_ of the sensor as a function of the applied pressure at 200 MHz.

By applying the lumped element equivalent circuit, which was described earlier, it was possible to separate the impedance change of the diaphragm metallization (FeSiB) *Z*_SI_ from the total *Z*_T_. The relation between *Z*_SI_ and the applied pressure is shown in Figure 9. It decreased until 12 kPa, and after that, higher pressures did not lead to a further change in impedance.

**Figure 10:**
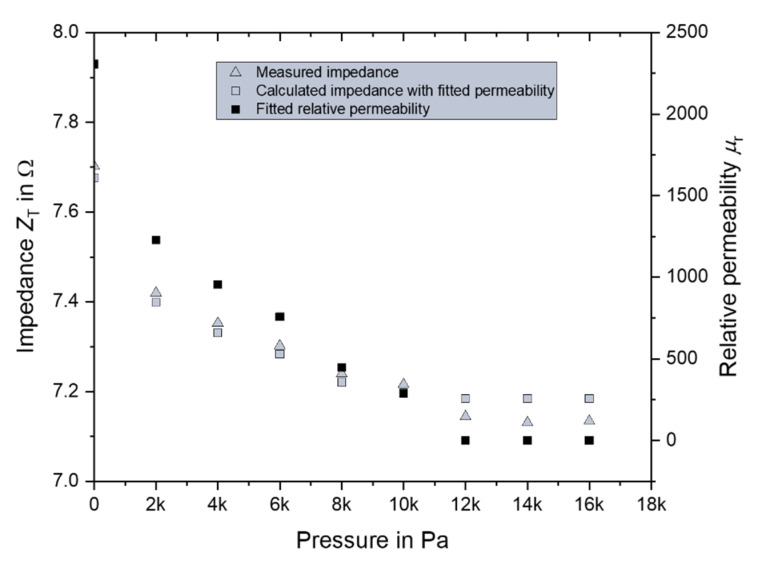
Relative permeability of the FeSiB film (right *y*-axis) obtained by fitting and a comparison of the measured impedance with that calculated with the obtained permeability.

By using this result and Equations (5)–(7), the permeability of the FeSiB for each pressure step was obtained by fitting with the Levenberg–Marquart method, as shown in Figure 10. The impedance was recalculated from the obtained permeability and was plotted on the same figure. It showed a good fitting with the measured impedance, indicating that the results were reasonable. At a pressure of 12 kPa, the relative permeability approached a value of 1, and the impedance did not vary greatly. The relationship between impedance and pressure was non-linear.

## 4. Discussion and Conclusions

The measurement results show a clear dependence of the impedance on the applied pressure. Additionally, it could be shown that the change in impedance can be observed by a change in the permeability of the soft magnetic FeSiB material. The applied pressure deforms the diaphragm and stress is created in the diaphragm. The stress causes a change in the magnetic anisotropy of the diaphragm material and, as a result, the permeability changes. The mechanical energy overcomes the domain wall energy and the domain wall moves. The measurement results show that, at a certain pressure, the impedance remains constant and the relative permeability approaches 1. We believe that this is because the magnetic material reached its saturation magnetization when its magnetic domain structure changed from multi domain to single domain under the induced stress. This is a limitation of the device.

To evaluate the usefulness of such a simple structure as a pressure sensor, we calculated the gauge factor *GF*, as in Equation (4). For this, the strain must be obtained. As the diaphragm is much thinner than its radius, the stress is approximated as being constant over the entire diaphragm. If the bending moments are small, the deflection is a function of the intrinsic stress and the elastic straining of the membrane. If the radial strain *ε*_r_ is assumed as being constant over the entire membrane, it can be estimated from the difference in the length of the diameter (2*r*) of an undeflected membrane and the segment of a parabola, corresponding to the cross-section of a membrane with a center deflection of *w*_0_, as follows:

(9) $$\varepsilon_{r}=\frac{2}{3}\frac{w_{0}^{2}}{r^{2}}$$

The strongest change in impedance was observed at 200 MHz. It is close to the electric resonance of the device where the reactance is at its minimum, reducing the influence of the parasitic elements. At 12 kPa pressure, the center deflection of the diaphragm *w*_0_ was 15.6 µm, and the transducer impedance *Z*_SI_ was 11.29 Ω. In the initial state (0 Pa), the impedance of the transducer was 15.07 Ω. By using Equations (4) and (9) [30], the result of *GF* = 385.7 could be obtained. The gauge factor was much higher than that of metal strain gauges (<10) and slightly higher than that of silicon strain gauges (~200). Although it was much lower than the optimized SI effect strain gauges, this very simple pressure sensor still demonstrates good potential for application. The sensitivity of the device is determined by the mechanical compliance of the diaphragm and the relationship between induced stress and impedance change. Mechanical compliance of a fully clamped circle diaphragm is defined as:

(10) $$w_{0}=\frac{3P\left(1-\vartheta^{2}\right)r^{4}}{16Et^{3}}$$

where *E* is the Young’s modulus, *ϑ* is the Poisson’s ratio, and *P* is the applied pressure. It can be seen that a thinner and larger diaphragm results in larger deflection at the same pressure. As a result, the strain and stress in the diaphragm are larger and the gauge factor should be higher. On the other hand, as shown in Equation (5), the impedance change of the diaphragm will be larger with higher *t* at the same *∆δ*, and if *t* << *δ*, the impedance change will be too small. Additionally, *t* and *r* of the diaphragm are also important for the maximum sustainable pressure. A diaphragm design should be optimized based on the sensor requirements.

**Figure 11:**
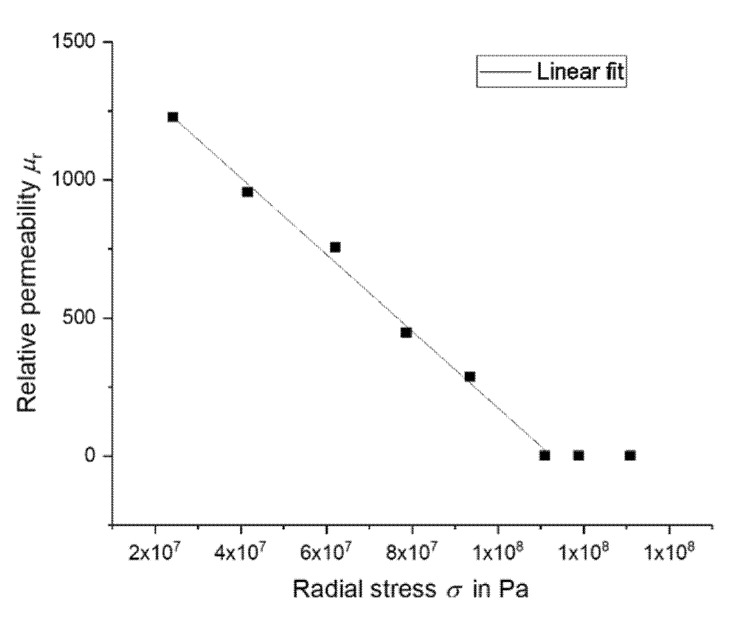
Relationship between relative magnetic permeability of the FeSiB and the radial stress in the diaphragm caused by deformation.

Under the assumption of Young’s modulus *E* = 170 GPa [31] and the strain obtained by Equation (9), it is possible to calculate the radial stress *σ* and to plot it against the relative permeability. Figure 11 shows this relationship.

Up to a relative permeability value approaching 1, it appeared to have a linear relation with the radial stress in the diaphragm. To confirm this relation, *µ*_r_ should be obtained directly by measurement in a vibrating-sample magnetometer (VSM) under different applied stresses in future experiments. In the related theory, it is known that magnetic permeability depends on the magnetic field acting on it [32]. However, a complete quantitative model of permeability as a function of mechanical stress has not yet been published. An advanced solution was proposed in reference [13]. The effective magnetic field in the material *H*_eff_ also considers the influence of mechanical stresses as linear:

(11) $$H_{eff}=H+\alpha M+\frac{3}{2}\frac{\lambda_{S}}{\mu_{0}M_{S}}\frac{M}{M_{S}}\sigma$$

*H* is the applied magnetic field, *M* is the magnetization of the material, and *α* is a dimensionless parameter representing interdomain coupling. The saturation magnetostriction *λ*_S_ may also depend on stress [33].

The impedance change of the diaphragm vs. the pressure change ($\;\frac{\Delta Z_{SI}}{∆P}$ ) was nonlinear, with a larger change at lower pressures. The reason is the also nonlinear mechanical behavior of the diaphragm at large deflections, as well as the complex relationship between *µ* and *Z*_SI_, as shown in Equations (2) and (5)–(7).

**Figure 12:**
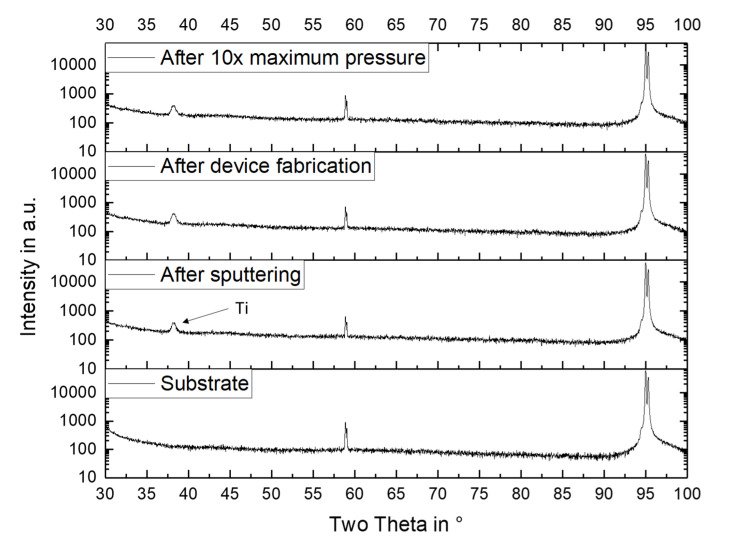
X-ray diffraction (XRD) pattern of the diaphragm metallization at different stages of fabrication and measurement, in comparison with the diffraction pattern of the Si substrate.

In this investigation, at a relative pressure of 40 kPa, a maximum stress of 250 MPa was applied to the diaphragm. It did not break or show any kind of damage. To obtain information about the structural stability of the diaphragm, its XRD pattern was recorded just after sputtering, after device fabrication, and after 10 times of applying of the maximum pressure to the device. Figure 12 shows the results. Beside the peaks resulting from the silicon substrate, Ti from the adhesion layer was detected after sputtering. There were no other sharp peaks detected, confirming that the FeSiB was in an amorphous state. After device fabrication and after applying the maximum possible pressure of 40 kPa, no apparent change in the diffraction pattern was observed. The impedance of the device also did not change. It can be concluded that the amorphous structure did not change during fabrication or under load.

It is known that amorphous FeSiB can have an extremely high tensile strength of more than 3.5 GPa [34]. This can sustain much larger strains than conventional strain transducers or fabricate very thin diaphragms with high mechanical compliance. Additionally, the transducer is just a simple diaphragm without structure or additional layers and can be fabricated easily. Typical micro machined pressure sensors using piezoresistive strain gauges, integrated into a silicon diaphragm. Beside the processes to fabricate the diaphragm itself, silicon piezoresistive strain gauges require several process steps, such as ion implantation and thermal diffusion, to create different doped regions in the semiconductor material of the diaphragm. By contrast, our proposed device only needs magnetron sputtering to form the diaphragm, as the diaphragm itself is already the transducer. Several lithography mask layers (to form the piezoresistive transducers in the diaphragm) are not needed. Furthermore, FeSiB, the material for the diaphragm, is not expensive and used for thin-film magnetic field sensors [2,7]. We believe that the proposed simple device has a good potential for use as a cheap sensor, with *GF* being at least as good as piezoresistive sensors. The influence of external electro-magnetic fields and other noise sources should be investigated in future work. To increase the magnetic anisotropy, permeability, and ultimately the *GF*, thermal annealing of the transducer metal, preferably in an external magnetic field, should be carried out in an attempt to improve the performance [35]. Another method is to apply a constant magnetic field during the deposition process.

## Figures and Tables

**Table 1:** Characteristic parameters of the sputtered FeSiB, obtained by superconducting quantum interference device (SQUID) measurement.

Parameters of Sputtered FeSiB	Measured Value
Saturation magnetic flux density *B*_s_	0.63 T
Coercivity *H*_c_	38 A/m
Saturation magnetization *M*_s_	5.03 × 10^5^ A/m
Remanent magnetization *M*_r_	2.34 × 10^4^ A/m

**Table 2:** Values of the element of the lumped equivalent circuit (Figure 6) obtained by fitting.

Lumped Equivalent Circuit Element	Fitted Value
*R*_1_	0.48 Ω
*R*_2_	0.50 Ω
*L*_1_	2.12 nH
*C*_1_	0.11 pF

## References

[1] Lamb H. (1883). On electrical motions in a spherical conductor. Philos. Trans. R. Soc. Lond..

[2] Yu J., Zhou Y., Cai B., Xu D. (2000). Giant magneto-impedance effect in amorphous magnetostrictive FeSiB thin films. J. Magn. Magn. Mater..

[3] Panina L.V., Mohri K. (1994). Mechanism of the Magneto-Impedance Effect in Negative Magnetostrictive Amorphous Wires. IEEE Transl. J. Magn. Jpn..

[4] Squire P.T., Atkinson D., Gibbs M.R.J., Atalay S. (1994). Amorphous wires and their applications. J. Magn. Magn. Mater..

[5] Machado F.L.A., Martins C.S., Rezende S.M. (1995). Giant magnetoimpedance in the ferromagnetic alloy Co75-xFexSi15B10. Phys. Rev. B.

[6] Hauser H., Kraus L., Ripka P. (2001). Giant magnetoimpedance sensors. IEEE Instrum. Meas. Mag..

[7] Mohri K., Uchiyama T., Shen L.P., Cai C.M., Panina L.V., Honkura Y., Yamamoto M. (2002). Amorphous wire and CMOS IC-based sensitive micromagnetic sensors utilizing magnetoimpedance (MI) and stress-impedance (SI) effects. IEEE Trans. Magn..

[8] Zhukova V., Blanco J.M., Ipatov M., Gonzalez J., Churyukanova M., Zhukov A. (2018). Engineering of magnetic softness and giant magnetoimpedance effect in Fe-rich microwires by stress-annealing. Scr. Mater..

[9] Nishibe Y., Yamadera H., Ohta N., Tsukada K., Nonomura Y. (2000). Thin film magnetic field sensor utilizing Magneto Impedance effect. Sens. Actuators A Phys..

[10] Semirov A.V., Bukreev D.A., Moiseev A.A., Kudryavtsev V.O., Derevyanko M.S. Influence of thermo-stress factor on magnetoimpedance of soft magnetic materials. Proceedings of the 2010 11th International Conference and Seminar on Micro/Nanotechnologies and Electron Devices.

[11] Gazda P., Nowicki M., Szewczyk R. (2019). Comparison of stress-impedance effect in amorphous ribbons with positive and negative magnetostriction. Materials.

[12] Harrison E.P., Rowe H. (1938). An impedance magnetometer. Proc. Phys. Soc..

[13] Sablik M.J., Kwun H., Burkhardt G.L., Jiles D.C. (1987). Model for the effect of tensile and compressive stress on ferromagnetic hysteresis. J. Appl. Phys..

[14] Kaviraj B., Ghatak S.K. (2007). Simulation of stress-impedance effects in low magnetostrictive films. J. Non. Cryst. Solids.

[15] Shen L.P., Uchiyama T., Mohri K., Kita E., Bushida K. (1997). Sensitive stress-impedance micro sensor using amorphous magnetostrictive wire. IEEE Trans. Magn..

[16] Fosalau C., Damian C., Zet C. (2013). A high performance strain gage based on the stressimpedance effect in magnetic amorphous wires. Sens. Actuators A Phys..

[17] Tejedor M., Hernando B., Sánchez M., Prida V., Vázquez M. (2000). Magneto-impedance effect in amorphous ribbons for stress sensor application. Sens. Actuators A Phys..

[18] Garcia-Arribas A., Combarro L., Goiriena-Goikoetxea M., Kurlyandskaya G.V., Svalov A.V., Fernandez E., Orue I., Feuchtwanger J. (2017). Thin-Film Magnetoimpedance Structures onto Flexible Substrates as Deformation Sensors. IEEE Trans. Magn..

[19] Suwa Y., Agatsuma S., Hashi S., Ishiyama K. (2010). Study on impedance change of strain sensor using magnetostrictive film. J. Magn. Soc. Jpn..

[20] Chen J.A., Ding W., Zhou Y., Cao Y., Zhou Z.M., Zhang Y.M. (2006). Stress-impedance effects in sandwiched FeCuNbCrSiB/Cu/FeCuNbCrSiB films. Mater. Lett..

[21] Hika K., Panina L.V., Mohri K. (1996). Magneto-impedance in sandwich film for magnetic sensor heads. IEEE Trans. Magn..

[22] Yamadera H., Nishibe Y. (2000). Strain-impedance properties of a CoSiB/Cu/CoSiB layered film. J. Appl. Phys..

[23] Mao X.H., Zhou Y., Chen J.A., Yu J.Q., Cai B.C. (2003). Giant magnetoimpedance and stress-impedance effects in multilayered FeSiB/Cu/FeSiB films with a meander structure. J. Mater. Res..

[24] Zhou Y., Ding W., Mao X.H., Chen J.A., Zhang Y.M., Gao X.Y. (2005). Stress-impedance effects in multilayered FeSiB/Cu/FeSiB films. Thin Solid Films.

[25] Vincent J.D.S., Rodrigues M., Leong Z., Morley N.A. (2020). Design and Development of Magnetostrictive Actuators and Sensors for Structural Health Monitoring. Sensors.

[26] Eaton W.P., Bitsie F., Smith J.H., Plummer D.W. A New Analytical Solution for Diaphragm Deflection and its Application to a Surface-Micromachined Pressure Sensor. Proceedings of the TechConnect Briefs: Tech. Proc. 1999 Int. Conf. Model. Simulations Microsystems.

[27] Mogilny G.S., Shanina B.D., Maslov V.V., Nosenko V.K., Shevchenko A.D., Gavriljuk V.G. (2011). Structure and magnetic anisotropy of rapidly quenched FeSiB ribbons. J. Non. Cryst. Solids.

[28] Landau L.D., Lifshitz E.M. (1984). Electrodynamics of Continuous Media.

[29] Hoer C., Love C. (1965). Exact inductance equations for rectangular conductors with applications to more complicated geometries. J. Res. Natl. Bur. Stand. Sect. C Eng. Instrum..

[30] Schomburg W.K., Goll C. (1998). Design optimization of bistable microdiaphragm valves. Sens. Actuators A Phys..

[31] Životský O., Titov A., Jirásková Y., Buršík J., Kalbáčová J., Janičkovič D., Švec P. (2013). Full-scale magnetic, microstructural, and physical properties of bilayered CoSiB/FeSiB ribbons. J. Alloys Compd..

[32] Jiles D. (2015). Introduction to Magnetism and Magnetic Materials.

[33] Bieńkowski A., Kulikowski J. (1991). Effect of stresses on the magnetostriction of Ni-Zn(Co) ferrites. J. Magn. Magn. Mater..

[34] Inoue A., Shen B.L., Chang C.T. (2006). Fe- and Co-based bulk glassy alloys with ultrahigh strength of over 4000MPa. Intermetallics.

[35] De-Ren L., Zhi-Chao L., Shao-Xiong Z., Jun-Feng Z., Hui L., Wei H. (2002). Giant Stress-Impedance Effect in Amorphous and High-Current-Density Electropulsing Annealed Fe73.5Cu1Nb3Si13.5B9 Ribbons. Chin. Phys. Lett..

